# Studies on Colony Stimulating Factor Receptor-1 and Ligands Colony Stimulating Factor-1 and Interleukin-34 in Alzheimer's Disease Brains and Human Microglia

**DOI:** 10.3389/fnagi.2017.00244

**Published:** 2017-08-09

**Authors:** Douglas G. Walker, Tiffany M. Tang, Lih-Fen Lue

**Affiliations:** ^1^Neurodegenerative Disease Research Center, Biodesign Institute, Arizona State University Tempe, AZ, United States; ^2^Laboratory of Neuroinflammation, Banner Sun Health Research Institute, Sun City Arizona, AZ, United States

**Keywords:** neuroinflammation, human microglia, neuropathology, quantitative polymerase chain reaction, RNA-sequencing, activation phenotype

## Abstract

Microglia are dependent on signaling through the colony stimulating factor-1 receptor (CSF-1R/CD115) for growth and survival. Activation of CSF-1R can lead to cell division, while blocking CSF-1R can lead to rapid microglia cell death. CSF-1R has two ligands, the growth factors colony stimulating factor-1 (CSF-1) and the more recently identified interleukin-34 (IL-34). Studies of IL-34 activation of rodent microglia and human macrophages have suggested it has different properties to CSF-1, resulting in an anti-inflammatory reparative phenotype. The goal of this study was to identify if the responses of human postmortem brain microglia to IL-34 differed from their responses to CSF-1 with the aim of identifying different phenotypes of microglia as a result of their responses. To approach this question, we also sought to identify differences between IL-34, CSF-1, and CSF-1R expression in human brain samples to establish whether there was an imbalance in Alzheimer's disease (AD). Using human brain samples [inferior temporal gyrus (ITG) and middle temporal gyrus (MTG)] from distinct cohorts of AD, control and high pathology, or mild cognitive impairment cases, we showed that there was increased expression of CSF-1R and CSF-1 mRNAs in both series of AD cases, and reduced expression of IL-34 mRNA in AD ITG samples. There was no change in expression of these genes in RNA from cerebellum of AD, Parkinson's disease (PD), or control cases. The results suggested an imbalance in CSF-1R signaling in AD. Using RNA sequencing to compare gene expression responses of CSF-1 and IL-34 stimulated human microglia, a profile of responses to CSF-1 and IL-34 was identified. Contrary to earlier work with rodent microglia, IL-34 induced primarily a classical activation response similar to that of CSF-1. It was not possible to identify any genes expressed significantly different by IL-34-stimulated microglia compared to CSF-1-stimulated microglia, but both cytokines did induce certain alternative activation-associated genes. These profiles also showed that a number of genes associated with lysosomal function and Aβ removal were downregulated by IL-34 and CSF-1 stimulation. Compared to earlier results our data indicate that CSF-1R stimulation by IL-34 or CSF-1 produced similar types of responses by elderly postmortem brain-derived microglia.

## Introduction

In the search for causes and treatments for neurodegenerative diseases such as Alzheimer's disease (AD), inflammation has been a major target. Although the identification of increased microglial activation associated with AD disease pathology was made more than 25 years ago, there are many aspects of neuroinflammation that still require investigations. The initial hypotheses that activated microglia in AD brains will be causing neurotoxicity by producing increased levels of damaging cytokines, reactive oxygen species, complement factors, and other potentially neurotoxic factors has not translated to effective therapies (Dickson et al., 1993; McGeer et al., 1993; Akiyama et al., 2000). Despite promise from epidemiological and experimental studies, many clinical trials of anti-inflammatory agents on patients at various stages of AD have not shown clinical efficacy (Aisen et al., 2003; Lyketsos et al., 2007; Meinert and Breitner, 2008). Many investigations of microglia in relation to disease have revealed essential functions needed to ensure brain homeostasis. The potential for manipulation of microglia to enhance amyloid beta (Aβ) phagocytosis through antibody or immunization as a therapeutic strategy has highlighted the other functions of microglia that need to be maintained (Zotova et al., 2011; Krabbe et al., 2013). Further support for key roles for inflammation and microglia came from genome wide association studies (GWAS) that identified single nucleotide polymorphisms (SNP) in microglial or inflammation-associated genes such as CD33, triggering receptor expressed by monocytic cells (TREM)-2, clusterin, and complement receptor-1 affecting the overall risk of AD (Kok et al., 2011; Chan et al., 2015; Hayden et al., 2015; Wang et al., 2017). In addition, apolipoprotein E (e4 variant), which can be expressed by microglia and is the strongest identified risk factor for sporadic AD, is also associated with enhanced inflammation. To understand the involvement of microglia in AD pathology relies on being able to identify activation phenotypes, but most studies have relied on the use of a restricted number of antigenic markers (e.g., HLA-DR, IBA-1, CD64, MSR-A, and CD68) combined with observations on microglial morphologies (Walker and Lue, 2015; Minett et al., 2016). Classification schemes for phenotyping macrophages developed to identify antigenic markers expressed in response to different classes of stimuli have been applied to studies of human brain microglia, but their applicability for human tissue studies has been questioned (Ransohoff, 2016).

A number of recent studies have considered the essential role of colony stimulating factor-1 receptor (CSF-1R) signaling in microglial maintenance and proliferation in normal and pathological conditions. CSF-1 was shown to be upregulated in AD and AD-like transgenic mice and was considered essential for the proliferation of microglia that occurs as a result of pathological activation in disease (Murphy et al., 2000; Vincent et al., 2002). Understanding the role of CSF-1R signaling changed with the identification of interleukin-34 (IL-34) as a CSF-1R ligand (Lin et al., 2008).

Human IL-34 is a 39 kDa protein in its native form and binds to the same receptor (CSF-1R) as CSF-1 (Lin et al., 2008; Chihara et al., 2010). Key findings have shown that there can be differences in microglia or macrophage responses to IL-34 compared to CSF-1 possibly due to the degree of phosphorylation of key signaling tyrosine residues on CSF-1R (Chihara et al., 2010; Mouchemore and Pixley, 2012). The main reason that this cytokine is of potential significance is that previous studies have indicated that IL-34 induces an anti-inflammatory phenotype in human monocytes/macrophages, where there was stronger induction of the anti-inflammatory cytokine IL-10 (Foucher et al., 2013). RNA sequencing analysis of IL-34 and CSF-1 responses by human blood identified that IL-34-treated cells had significantly less suppression of CCR2, the receptor for monocyte chemoattractant protein-1 (MCP-1) (Barve et al., 2013). Central to the premise of this study were findings that used cultured rodent microglia and animal models relevant to AD (Mizuno et al., 2011). IL-34 promoted microglial uptake and metabolism of Aβ, and as a consequence IL-34-treated microglia that had been stimulated with Aβ showed significantly lower neurotoxicity. The mechanism of action in this system appeared to be through transforming growth factor beta-1 (TGFβ-1) (Ma et al., 2012). IL-34 treated microglia showed increased levels of cell division and increased levels of TGFβ-1. Blocking TGFβ-1 receptor prevented IL-34 induced microglia cell division and prevented the reduction in neurotoxicity (Ma et al., 2012).

Key studies have shown how significant IL-34 is for development and maintenance of microglia in brain (Greter et al., 2012; Wang et al., 2012; Wang and Colonna, 2014). IL-34 appeared to be required for maintenance of microglia in adult brain, while CSF-1 was primarily involved in replacement of microglia after inflammation had occurred (Greter et al., 2012; Wang et al., 2012).

As a special role of IL-34 in brain has been suggested, we investigated IL-34 expression in human brains, and its effects on elderly brain-derived microglia compared to CSF-1. Our findings showed altered patterns of expression of IL-34 mRNA compared to CSF-1 and CSF-1R in AD-affected brains. Using human microglia stimulated with IL-34 or CSF-1, we could not detect any differential patterns of gene expression, but could define IL-34 and CSF-1 activation of human microglia as inducing a primarily proinflammatory phenotype. CSF-1R activation of human microglia by these ligands also appeared to result in downregulation of genes associated with amyloid removal. These results could be of significance for considering how inflammation could be manipulated in AD to reduce pathology.

## Materials and methods

### Human brain tissue resources

All brain samples were from participants in the Arizona Study of Aging and Neurodegenerative Disorders and were autopsied by the Brain and Body Donation Program (BBDP) (www.brainandbodydonationprogram.org) of the Banner Sun Health Research Institute, Sun City, Arizona. Details of this program can be found in these reviews (Beach et al., 2008, 2015). This longitudinal clinicopathological study has been running for 27 years with continuous Institutional Review Board (IRB) approval. Samples used in this study had been collected over a period of 18 years. During that time, the IRB was run internally by Sun Health Corporation (up to 2008), then internally by Banner Health Corporation (2008–2011), and then on contract by external agency Western IRB (Olympia, WA). Use of the human pathological samples from the BBDP were provided to internal and external researchers with no identifiable information, and as such is not considered human subject research under exemption 4 of regulations 45 CFR 46.101(b). All cases were diagnosed according to National Institutes on Aging/Reagan criteria for AD (Newell et al., 1999). A summary of the demographics of all of the cases used in this study are shown in Tables 1A-D. A series of samples derived from inferior temporal gyrus (ITG) from non-demented controls (ND), mild cognitive impaired (MCI), and AD were used initially (Table 1A); a second series of samples included for validation were derived from middle temporal gyrus (MTG) of ND (low-plaque cases), ND (high-plaque cases), and AD subjects (Table 1B). A series of cerebellum samples were also analyzed as a control region that generally has minimal AD pathology (Table 1C). The cerebellum samples were from ND, AD, and Parkinson's disease (PD) cases. These series of samples were used for gene expression experiments. A separate series of ITG samples from ND and AD cases were used for western blot analyses (Table 1D).

**Table 1:** Demographic details of human brain cases used.

**Disease state (*n*)**	**Age**	**Sex**	**PMI (hrs)**	**ApoE4 (%)**	**Plaques**	**Tangles**
**A: INFERIOR TEMPORAL GYRUS (RNA EXPRESSION)**						
ND (*n* = 12)	85.2 ± 7.8	9M/3F	2.8 ± 1.0	4	2.7 ± 3.8	3.5 ± 1.6
MCI (*n* = 13)	88.5 ± 6.7	10M/3F	2.8 ± 0.8	23	9.8 ± 3.1	5.9 + 2.6
AD (*n* = 12)	79.6 + 8.4	5M/7F	3.3 ± 1.0	21	13.8 ± 1.7	14.2 ± 1.7
**B: MIDDLE TEMPORAL GYRUS (RNA EXPRESSION)**						
LPND (*n* = 14)	85.4 ± 9.0	7M/7F	3.1 ± 1.0	4	1.1 ± 1.8	5.3 ± 2.4
HPND (*n* = 13)	87.3 + 7.1	6M/7F	2.7 ± 0.3	11.5	11.4 ± 1.9	5.1 ± 2.0
AD (*n* = 15)	79.7 + 4.6	10M/5F	3.5 ± 0.6^*^	30	14.3 ± 0.8	13.4 ± 2.4
**C: INFERIOR TEMPORAL GYRUS (WESTERN BLOT)**						
ND (*n* = 25)	84.0 ± 6.3	16M/9F	2.9 ± 0.9	12	3.1 ± 4.3	3.8 ± 2.4
AD (*n* = 16)	77.8 + 11.6	8M/8F	3.1 ± 1.3	44	13.0 ± 1.3	12.9 ± 2.4
**D: CEREBELLUM (RNA EXPRESSION)**						
ND (*n* = 10)	88.8 ± 9.25	7M/3F	3.84 ± 2.1	0	1.9 ± 3.0	5.5 ± 2.4
AD (*n* = 14)	82.9 ± 11.9	7M/7F	3.35 ± 0.9	32	13.9 ± 1.0	13.3 ± 2.2
PD (*n* = 13)	77.1 ± 6.9	10M/3F	3.66 ± 1.4	11.5	0.4 ± 0.8	5.8 ± 3.5
**E: FRONTAL CORTEX (MICROGLIA CULTURE)**						
2ND/6AD (*n* = 8)	84.4 + 5.9	4M/4F	3.4 ± 0.8	37.5		

* *Significant difference (P < 0.05) between HPND and AD groups. Abbreviations: PMI, post mortem interval; ND, non demented; MCI, Mild Cognitive Impaired; AD, Alzheimer's Disease; LPND, low plaque non-demented; HPND, high plaque non-demented*.

As part of the diagnostic process, each brain was assessed for plaque and tangle load using histological procedures. This ranking method gives a score (0–3) for each of five brain regions (entorhinal cortex, hippocampus, temporal cortex, parietal cortex, and frontal cortex) for a potential summary score (0–15) and is based on the histological assessment of frequency of plaques and tangles in Thioflavin S-stained tissue sections (Beach et al., 2012).

For isolation of human microglia, samples of frontal cortex from eight separate cases were used. These were provided within 3 h of death by the BBDP. Tissue was provided at time of autopsy from donors consented to participate in the BBDP autopsy program. The BBDP program consent form provides approval for supplying tissue to approved researchers both internally and externally for different projects. The tissue was provided to researchers for microglia culture without any personal identifiable information, and as such meets the requirements for exemption 4 for human subject research. The demographic details of the cases used for microglia culture are listed in Table 1E.

### RNA isolation and quantitative real time polymerase chain reaction

RNA was prepared from human brain tissue samples, and cultured human microglia using RNAeasy Plus Mini kits (Qiagen, Valencia, CA) according to the manufacturer's directions with integrity assessed with an Agilent Bioanalyzer and RNA 6000 Nano kits (Agilent, Santa Clara, CA). Samples used for qPCR had RIN > 7.0, and those used for RNA-seq had RIN > 8.0. Samples which did not meet this criteria were excluded from the study. RNA from brain samples (0.5 μg) and cultured cell samples (0.2 μg) were reverse transcribed using the Quantitect reverse transcription kit (Qiagen). Appropriate numbers of no reverse transcriptase controls were prepared in parallel for each batch of samples. For qPCR, cDNA samples were amplified using Perfecta Fast Mix 2x reaction mixture (Quanta Biosciences, Gaithersburg, MD) supplemented with 1.25 μM of EvaGreen. The primers used to detect CSF-1R (CD115), CSF-1, IL-34, Transcription factor EB (TFEB), CD68, IL-1β, and β actin are listed in Table 2. QPCR was carried out using a Stratagene Mx3000p machine and abundance of gene expression quantified relative to a standard curve. All PCR-values were normalized against values for β-actin mRNA expression as described previously (Walker et al., 2009, 2015). QPCR analyses followed most recommended criteria for minimal information for publication of quantitative real time PCR experiments (MIQE) (Bustin et al., 2009).

**Table 2:** PCR primer sequences.

	**Sequence**	**Amplicon (bp)**	**Ref. Seq**.
CSF-1R sense	GCACCAACAACGCTACCT	147	NM_005211.3
CSF-1R antisense	CGAACACGACCACCTCCT		
CSF-1 sense	ACCCCTCCACCCTCTCTG	133	NM_000757.5
CSF-1 antisense	CTGCCCCTTCACTTGCTG		
IL-34 sense	TTGACGCAGAATGAGGAGTG	100	NM_005211.3
IL34 antisense	GTTGATGGGGAAGTAGTGTTTG		
IL-1β sense	CTGTCCTGCGTGTTGAAAGA	180	NM_00576.2
IL-1β antisense	TTCTGCTTGAGAGGTGCTGA		
TFEB sense	AGCAGGTGGTGAAGCAGGAG	154	NM_007162.2
TFEB antisense	AGGTGATGGAATGGGGATG		
CD68 sense	GCTACTTTGCTGCCATCCTT	103	NM_001251.2
CD68 antisense	TCCTGTGAGTGGTGGTTTTG		
β actin sense	TCCTATGTGGGCGACGAG	242	NM_001101.3
β actin antisense	ATGGCTGGGGTGTTGAAG		

### Western blot analysis

Protein extracts from temporal cortex or microglia were analyzed by western blot methodology for levels of IL-34 protein using our published protocols (Walker et al., 2015, 2009) Samples were dissolved at a concentration of 1 μg/μl protein in western blot sample buffer (NUPAGE LDS—Life Technologies, Carlsbad, CA) containing 0.1 M DTT and heated at 70°C for 10 min. Samples were separated on 4–12% NuPAGE Bis–Tris Mini gels using MOPS or MES running buffer (Life Technologies). Proteins were transferred to nitrocellulose membranes at 30 V for 90 min, which were blocked in 5% skim milk solution dissolved in Tris-buffered saline [TBST—50 mM Tris–HCl (pH 8.0), 250 mM NaCl, 0.05% (w/v) Tween 20], and then reacted for 18 h in appropriate dilutions of antibodies in TBST containing 2% milk. Bound antibodies were detected by reaction for 2 h with the appropriate horseradish peroxidase (HRP) labeled anti-immunoglobulin (Thermo-Fisher—1:10,000 dilution) followed by reaction of membranes with HRP chemiluminescent substrate (Advansta Western blot Bright chemiluminescent substrate, Advansta, Menlo Park, CA) with direct imaging using a FluorochemQ imaging system (Protein Simple, San Jose, CA). Intensities of chemiluminescent bands were quantified using Fluorochem Q SA software (Protein Simple). Three different antibodies were used for detection of IL-34; a IL-34 sheep polyclonal (R&D Systems, Cat. No. AF5265), a rabbit polyclonal (Abcam, Cambridge, MA, Cat. No. ab75723), and a mouse monoclonal antibody (Abcam, Cat. No. ab101443). Western blots were reprobed to detect β-actin for normalization purposes (mouse monoclonal: 1:5,000, Sigma (St. Louis, MO). Validation of antibodies used HEK cells transfected with plasmids either to green fluorescent protein (Genecopoeia—Catalog number EX-EGFP-Lv105) or IL-34 (Genecopoeia—Catalog number EX-H9354-Lv105 in pReceiver-Lv105 plasmid) http://www.genecopoeia.com/product/search/detail.php?prt=1&cid=&key=H9354) (**Figure 2**). This figure illustrates results for the rabbit polyclonal to IL-34 (Abcam), but similar results were obtained for each IL-34 antibody.

### RNA-seq protocol

RNA samples analyzed were from four separate human microglia cases and one sample of human blood-derived macrophages that were either unstimulated (peptide diluent), or stimulated with IL-34 (100 ng/ml, Cat. No. 5265-IL-010, R&D Systems, MN) or CSF-1 (100 ng/ml, Cat. No. 216-MC-005, R&D Systems, MN) for 24 h. All of these microglia cases were isolated from subjects that had clinical and neuropathological diagnoses of AD or probable AD. For these experiments, 2 × 10^5^ microglia per well were plated out and stimulated with the indicated doses. All RNA samples used for RNA seq analyses had RIN > 8.0. RNA-seq analyses were carried out at the Translational Genomics Research Institute, Phoenix, AZ. Next Gen RNA sequencing was carried out using Illumina Hiseq 2000 platform. The mRNA libraries were prepared from each sample using Illumina RNA sample prep kits following previously described protocols (Henderson-Smith et al., 2016). Clusters were generated on Paired End v3 flowcells in the Illumina cBot using Illumina's TruSeq PE Cluster Kit v3, which were sequenced by synthesis on the Illumina HiSeq 2000 for paired 100-bp reads. Due to the small number of samples, data analysis was restricted to estimation of FPKM (fragments per kilobase of exon per million fragments mapped), a digital count of each transcript adjusted for its overall size using TopHat/Cufflinks/Cuffdiff software. The FPKM results were used to evaluate differential expression of genes between treatments. The mean FPKM-values for each of the four separate microglia samples were selected for genes of interest.

### Human brain cultures and experimental treatments

Human autopsy brain microglia were isolated from frontal cortex according to our standard protocols (Walker et al., 2006, 2009, 2015). After isolation, microglia were cultured for 10–14 days prior to use in experiments. Microglia isolated from eight separate cases were used in this study including the four cases used for RNA seq. We also isolated human brain endothelial cells from digested brain material by selection with *Ulex Europaeus* (UEA)-conjugated magnetic beads (Life Technologies).

### Statistical analysis

All statistical analyses were performed using Graphpad Prism version 7 (Graphpad software, San Diego, CA). One-way ANOVA followed by the Fisher LSD-test for *post-hoc* comparison between groups was used to demonstrate treatment or disease group differences. Correlation analyses used the Spearman method for non-parametric measures was carried out to determine relation between plaque and tangle scores and gene expression measurements. The significance level was defined as *P* < 0.05.

## Results

### Altered mRNA expression of CSF-1R, CSF-1, and IL-34 in human AD brain samples

**Figure 1:**
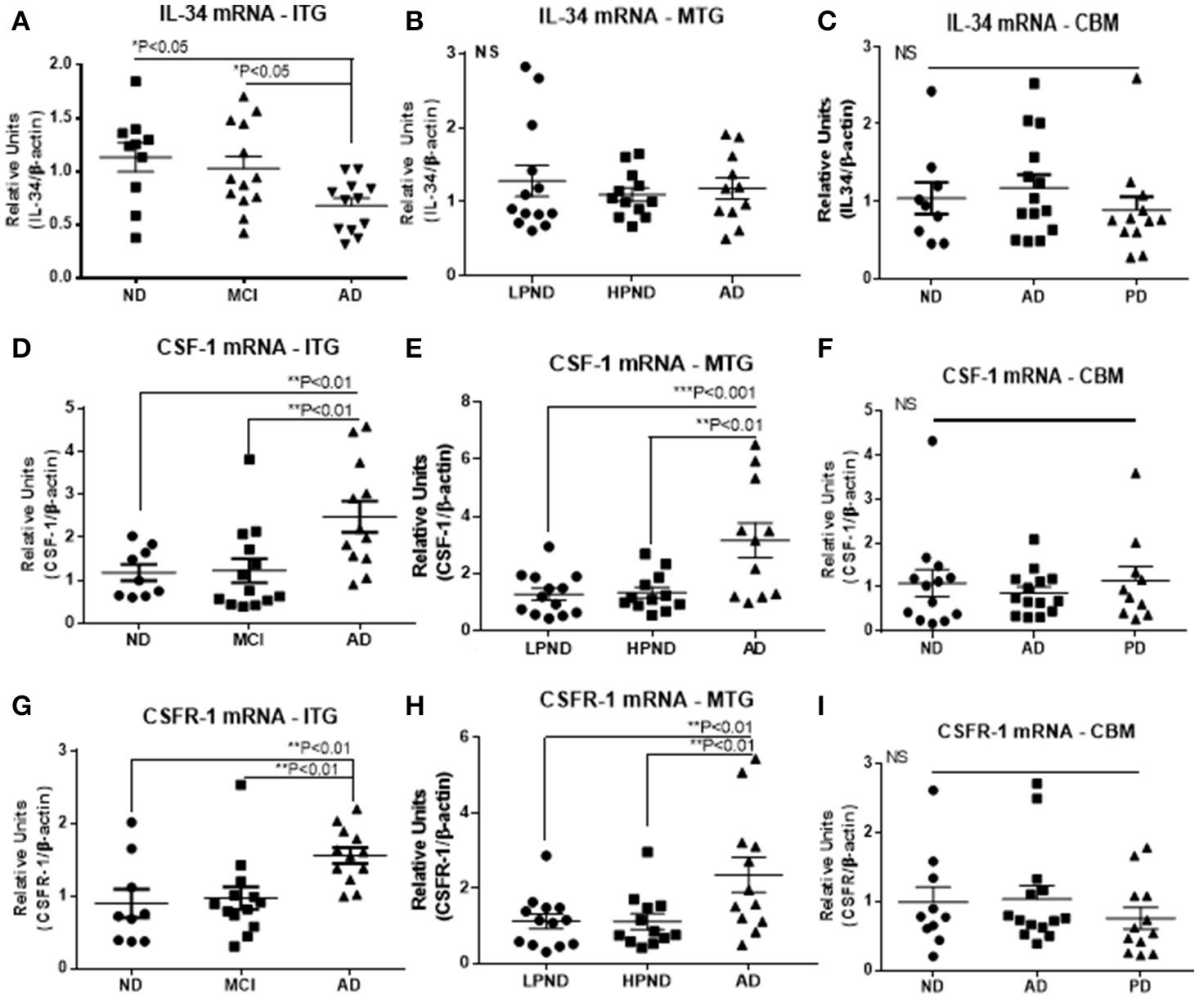
Scatter plots for qPCR analyses of IL-34, CSF-1, and CSF-1R expression levels in inferior temporal gyrus (ITG), middle temporal gyrus (MTG), and cerebellum (CBM) brain RNA samples. Results on left side of figure show relative expression of IL-34 **(A)**, CSF-1 **(D)**, and CSF-1R **(G)** in ITG RNA samples from non-demented (ND), mild cognitive impairment (MCI), and Alzheimer's disease (AD) cases. Results on figures show expression of IL-34 **(B)**, CSF-1 **(E)**, and CSF-1R **(H)** in MTG RNA samples from low plaque non-demented (LPND), high plaque non-demented (HPND), and Alzheimer's disease (AD) cases. Results on right side of figure show relative expression of IL-34 **(C)**, CSF-1 **(F)**, and CSF-1R **(I)** in cerebellum RNA samples from ND, AD, and Parkinson's disease (PD) cases. Data were analyzed by one-way ANOVA with Fisher LSD *post-hoc* test for between group significance. Bars on figures indicate mean ± standard error of mean (SEM) for each analyses.

Two series of human brain tissue samples were used to assess the relative changes in expression of mRNA of CSF-1R and its ligands CSF-1 and IL-34 with progression of disease. The first series from ITG (Table 1A) contained samples separated into groups depending on cognitive diagnosis, namely non-demented (ND), mild cognitive impairment (MCI), and AD dementia. The MCI group had intermediate levels of neuropathology between ND and AD. The second series from MTG (Table 1B) contained samples separated into groups depending on amount of plaque pathology, namely low plaque-non-demented (LPND), high plaque-non-demented (HPND), and AD. There was a progressive increase in AD-type plaque and tangle pathology between the groups. In the ITG group, there was a significant increase in mRNA levels of CSF-1R and CSF-1 in the AD samples compared to ND or MCI samples (Figure 1), but a significant decrease in IL-34 mRNA levels. The increased expression of CSF-1 and CSF-1R in AD was replicated in MTG samples, but there was not the decrease in IL-34 expression in this group of samples (Figure 1). For comparison, a group of cerebellum samples from ND, PD, and AD cases were used (Figure 1, Table 1C); this is a brain region not normally affected by neuropathology. There was no changes in expression of any of these genes in cerebellum.

Non-parametric (Spearman) correlation analyses were carried out with both series of data from cortical samples to determine if there was association between expression levels and degree of plaque or tangle pathology (Table 3). Data show no correlation between IL-34 mRNA expression and degree of AD pathology, while there were significant correlations for CSF-1R mRNA expression with plaque and tangle scores for both brain regions. Similarly, significant correlation was seen for CSF-1 mRNA expression and tangles in both brain regions, and significant correlation with plaque scores in MTG. The correlation between CSF-1 and plaque for ITG was close to significance (*P* = 0.064) (Table 3). Overall, these data suggest that CSF-1 and CSF-1R genes are regulated in different manners compared to IL-34.

**Table 3:** Correlations of IL-34, CSF-1, and CSF-1R mRNA expression with plaque and tangle pathology.

	**Plaques**	**Tangles**
**INFERIOR TEMPORAL GYRUS**		
IL-34	*r* = −0.07	*r* = −0.02
	*P* = 0.69	*P* = 0.91
CSF-1	*r* = 0.33	***r* = 0.35**
	*P* = 0.064	^*^P = 0.042
CSF-1R	***r* = 0.39**	***r* = 0.44**
	^*^P = 0.02	^**^P = 0.009
**MIDDLE TEMPORAL GYRUS**		
IL-34	*r* = 0.14	*r* = 0.18
	*P* = 0.43	*P* = 0.28
CSF-1	***r* = 0.44**	***r* = 0.35**
	^**^P = 0.007	^*^P = 0.036
CSF-1R	***r* = 0.37**	***r* = 0.34**
	^*^P = 0.02	^*^P = 0.04

Bold type represents statistically significant results;

* P < 0.05;

** *P < 0.01*.

### Reduced levels of IL-34 protein in AD brains

**Figure 2:**
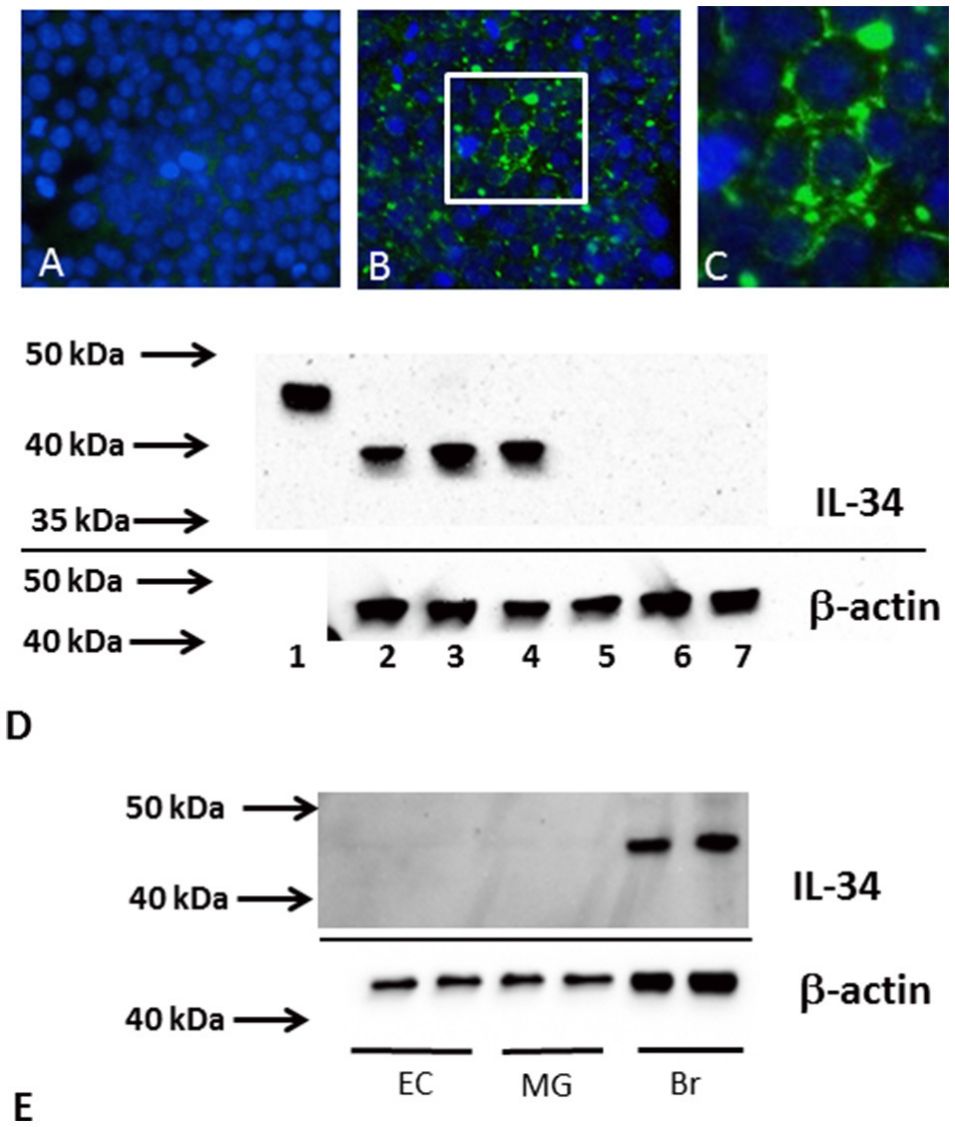
IL-34 antibody validation. Results shown employed a rabbit polyclonal to IL-34 (Abcam no. AB74548). Similar results were obtained using mouse monoclonal to IL-34 (AB101443) and sheep polyclonal to IL-34 (R and D Systems, AF5265). **(A–C)** Immunocytochemistry staining of HEK cells transfected with control plasmid (GFP) **(A)** or plasmid coding IL-34 **(B,C)**. Specific immunostaining (green) was only detected with IL-34 plasmid. Cells were counterstained with DAPI (blue). Low magnification **(B)** and higher magnification **(C)**. **(D)** Western blot showing detected bands using AB74548 antibody on recombinant IL-34 (lane 1), and different IL-34 transfected HEK cell samples (lanes 2–4), and different GFP plasmid transfected HEK cell samples (lanes 5–7). The cell expressed IL-34 had molecular weight of approximately 39 kDa. **(E)** Western blot showing a single band (45 kDa) detected in human brain samples (Br) but not in microglia (MG) or endothelial cell (EC) samples.

**Figure 3:**
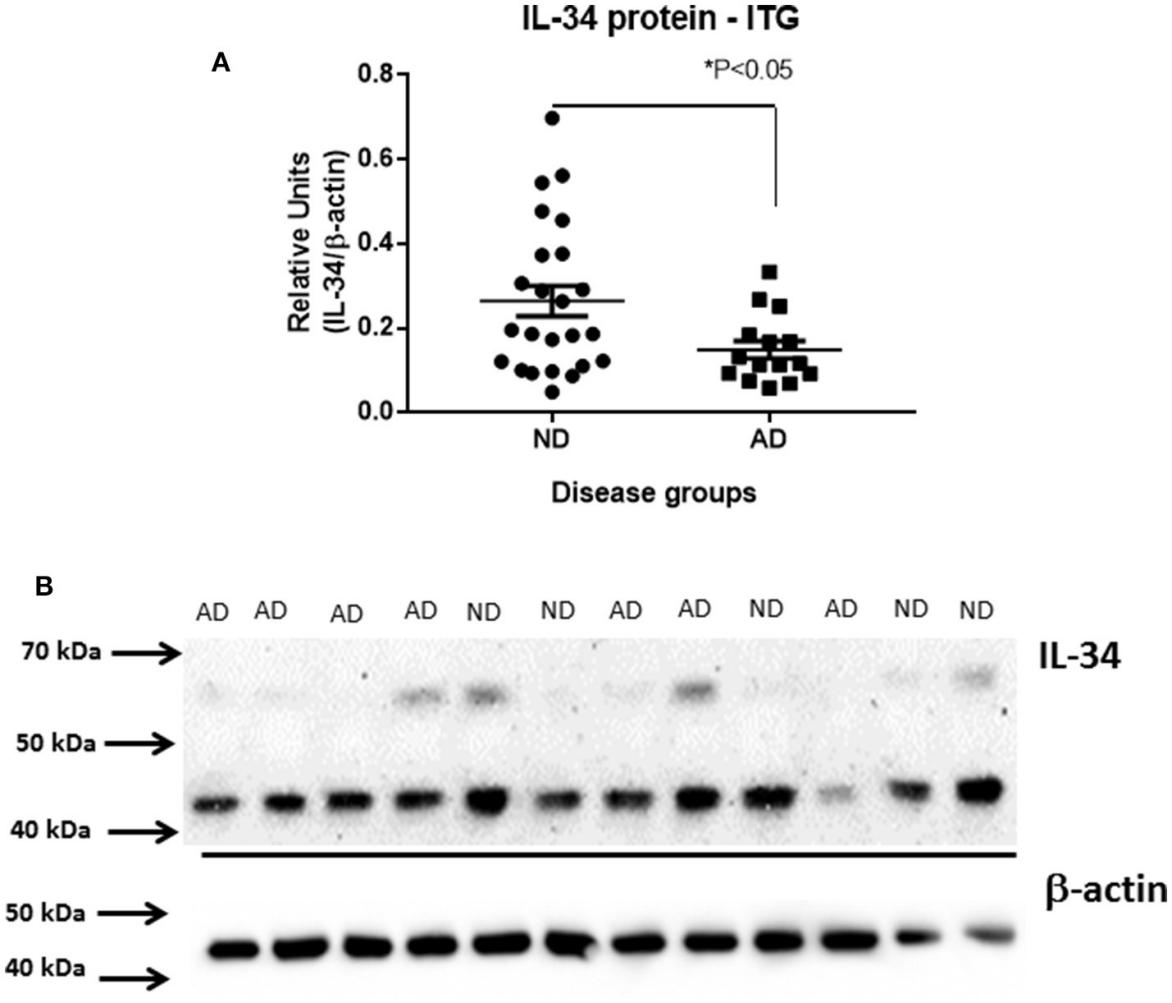
Decreased levels of IL-34 protein in ITG samples of AD cases compared to ND cases. **(A)** Scatter plot showing relative levels of IL-34 protein, detected with AB74548 rabbit polyclonal antibody. Significant decrease (*P* < 0.05) in IL-34 protein levels in AD cases. **(B)** Representative western blot of IL-34 protein detected in a range of brain samples analyzed in **(A)**. A major band of 45 kDa was detected but an additional band of approximately 60 kDa was detectable with longer exposure.

Using protein extracts from ITG tissue from a series of ND (*n* = 25) and AD cases (*n* = 16), western blot analyses using the rabbit polyclonal antibody to IL-34 were carried out (validation of this antibody is shown in Figure 2). The molecular weight of IL-34 in human brain (45 kDa) differed from that of recombinant plasmid expressed IL-34 (approximately 39 kDa) possibly due to different degrees of glycosylation of IL-34 expressed in HEK cells. In human brain samples, a weaker band of approximately 60 kDa was also present. Our results showed a significant decrease in IL-34 levels (45 kDa band) in the AD ITG samples (*P* < 0.05; Figure 3).

### Effect of IL34 and CSF-1 on microglial phenotypes: gene expression analysis

Human brain-derived microglia isolated from four different human cases were used to investigate the effects of CSF-1R ligands on microglial gene expression. Each microglia isolate was processed under identical conditions of culture, stimulation, and RNA-seq analyses. The main aims of these experiments were to identify any genes differentially expressed by IL-34 compared to CSF-1 treatment, and also to define the phenotype of human microglia exposed to the CSF-1R ligands. Data analyses of gene expression of all genes showed no genes were differentially expressed in IL-34 compared to CSF-1 stimulated microglia. The following strategies were adopted to define the phenotype of CSF-1R activation. Using panels of proinflammatory and anti-inflammatory markers taken from two key papers on phenotyping of activated, we extracted data of the relative changes between control and CSF-1R ligand-treated microglia for 31 proinflammatory and 33 anti-inflammatory (alternative activation) markers to assess the number of genes that were upregulated, unchanged or downregulated (Martinez et al., 2006; Murray et al., 2014). Of the selected anti-inflammatory genes, five were found not to be expressed by human microglia (ALOX15, CCL17, CD200R, TG, P2RY14). These result are shown in Table 4. The data showed that 18 of 31 proinflammatory-associated genes were upregulated, 8 were unchanged and 5 were downregulated. By comparison, 10 of the anti-inflammatory genes were upregulated, 5 were unchanged, and 13 downregulated. As we considered that the downregulated expression of anti-inflammatory genes were an indication of proinflammatory activation, combining these results showed 31 of the gene expression changes were indicative of CSF-1R ligands inducing a proinflammatory phenotype, while 15 of the gene expression changes were associated with an anti-inflammatory phenotype. Table 4 shows the separate data of stimulation indices for IL-34 and CSF-1 along with the mean of the combined data. These data for this restricted gene set confirmed that there was no difference between IL-34 and CSF-1 in cellular gene responses as was shown for the complete dataset. The predominant increase in expression of the classical proinflammatory cytokines IL1B, TNF, IL6, IL8, and IL1A and downregulation of SEPP1 and TLR5 strongly support the conclusion of IL-34 and CSF-1 produce primarily a proinflammatory response in microglia.

**Table 4:** Relative Levels of expression of genes designated to correlate with proinflammatory or anti-inflammatory phenotype in microglia groups following treatment with interleukin-34 (IL34) or colony stimulating factor-1 (CSF-1).

	**IL-34**	**CSF-1**	**Mean**
**PROINFLAMMATORY PHENOTYPIC MARKERS**			
**Upregulated (18)**			
IL1B	19.3	14.3	16.8
TNF	3.1	3.1	3.1
MARCO	2.5	3.2	2.9
IL6	3.3	2.0	2.6
IL8	2.9	1.7	2.3
CXCL10	2.6	1.7	2.1
IL1A	2.0	1.7	1.9
TLR2	1.8	1.7	1.6
CXCL11	1.8	1.4	1.6
SPHK1	1.5	1.6	1.6
PSME2	1.6	1.5	1.5
IDO1	2.1	0.9	1.5
IL23A	1.5	1.3	1.4
CXCL9	1.7	1.1	1.4
IRF7	1.4	1.3	1.4
BCL2AI	1.4	1.3	1.3
IL27	1.6	1.0	1.3
STAT1	1.4	1.2	1.3
**Unchanged (8)**			
IL15	1.0	1.1	1.1
IRF1	1.1	1.0	1.0
CCL5	1.1	1.0	1.0
APOL2	1.1	1.0	1.0
CD40	1.1	1.0	1.0
PTX3	1.0	1.0	1.0
KYN	1.0	0.9	1.0
IL15RA	1.0	0.9	1.0
**Downregulated (5)**			
IGFBP4	0.9	0.9	0.9
CCL18	0.8	0.8	0.8
IL12A	0.8	0.8	0.8
IRF5	0.8	0.8	0.8
CCR7	0.5	0.6	0.5
**ANTI-INFLAMMATORY PHENOTYPIC MARKERS**			
**Upregulated (10)**			
MMP12	1.7	1.6	1.7
CCL4	1.9	1.4	1.7
CD209	1.5	1.5	1.5
SOCS3	1.5	1.2	1.4
TGFB1	1.4	1.3	1.3
MRC1	1.1	1.5	1.3
TGM2	1.4	1.2	1.3
MSR1	1.2	1.2	1.2
IL1RN	1.1	1.2	1.1
ADORA3	1.1	1.1	1.1
**Unchanged (5)**			
IL4R	1.1	1.0	1.0
CD163	1.0	1.0	1.0
IL17RB	0.9	1.0	1.0
CTSC	1.0	0.9	1.0
SOCS1	1.1	0.8	1.0
**Downregulated (13)**			
CA2	0.9	0.9	0.9
FN1	0.9	0.9	0.9
MMP1	0.8	0.9	0.8
LIPA	0.8	0.9	0.8
CCL18	0.8	0.8	0.8
ID3	0.8	0.7	0.8
HEXB	0.7	0.8	0.7
CCL13	0.9	0.6	0.7
CD36	0.6	0.8	0.7
IRF4	0.7	0.6	0.7
SEPP1	0.6	0.6	0.6
RGS1	0.6	0.6	0.6
TLR5	0.4	0.4	0.4

**Figure 4:**
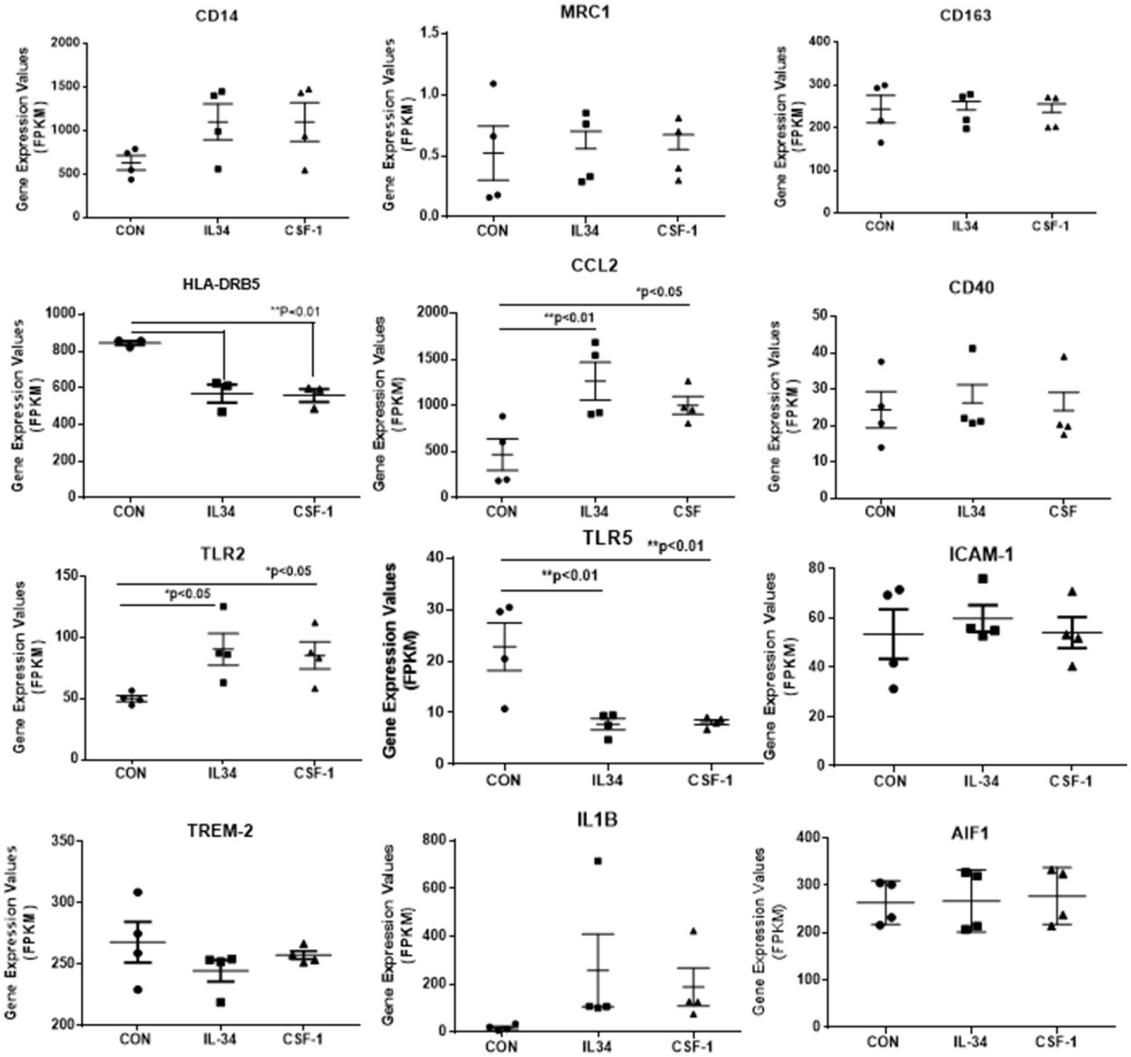
Scatter plots showing levels of expression of transcripts (FPKM) of key markers of activation and function expressed in control, IL-34, and CSF-1-stimulated human brain microglia (*n* = 4) for each treatment. Results were obtained by RNA sequencing. Due to the small number of cases, there was not statistically significant difference between groups in all cases, but many of the selected genes show trends for upregulation or downregulation of expression consistent with an altered phenotype of microglia. Data were analyzed by one-way ANOVA with Fisher LSD *post-hoc* test for between group significance. Bars on figures indicate mean ± standard error of mean (SEM) for each analyses. Significant changes were shown for expression of HLA-DRB5, CCL2, TLR2, and TLR5.

**Figure 5:**
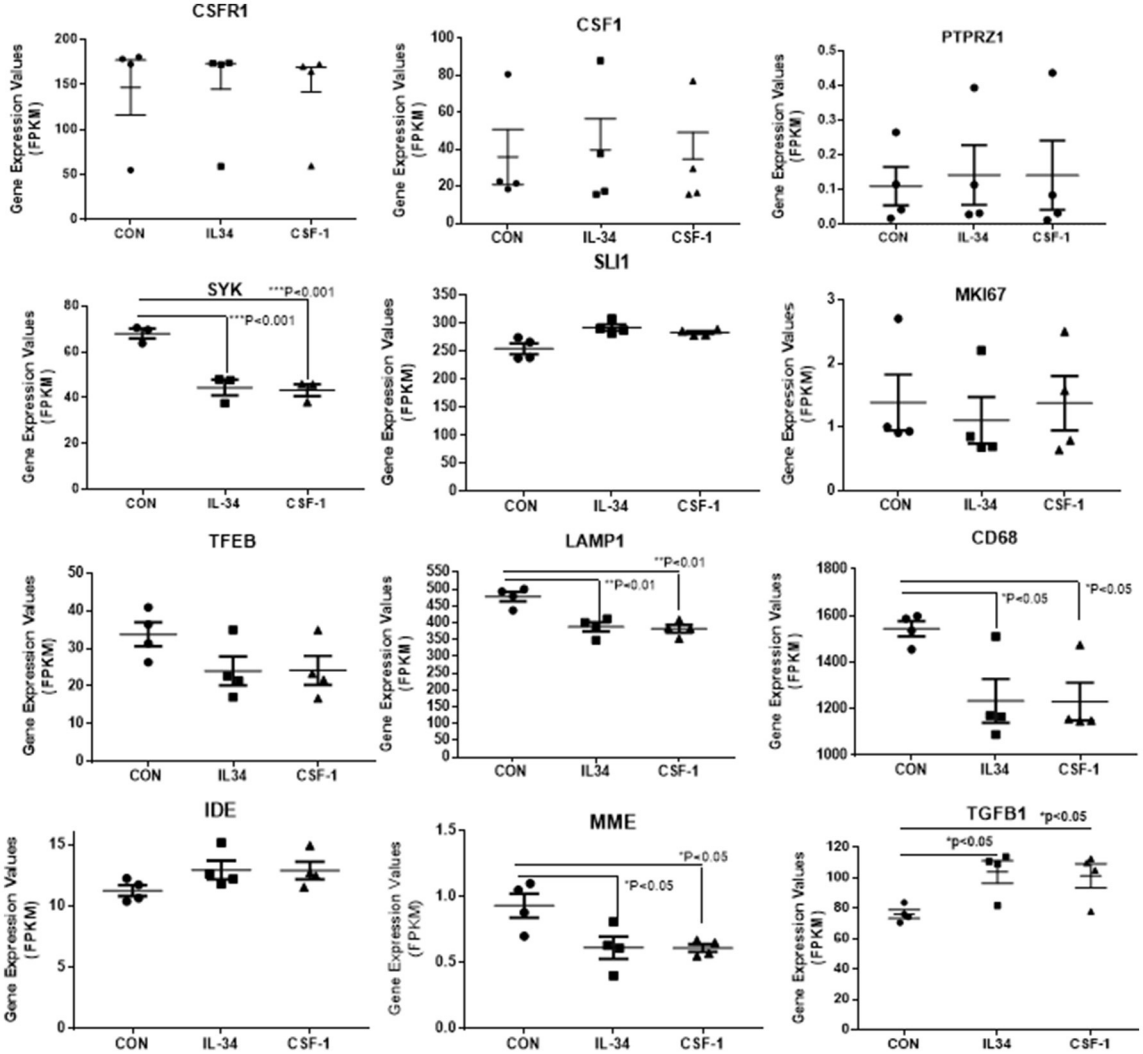
Scatter plots showing levels of expression of transcripts (FPKM) of key markers of activation and function expressed in control, IL-34, and CSF-1-stimulated human brain microglia (*n* = 4) for each treatment. Results were obtained by RNA sequencing. Due to the small number of cases, there was not statistically significant difference between groups in all cases, but many of the selected genes show trends for upregulation or downregulation of expression consistent with an altered phenotype of microglia. Data were analyzed by one-way ANOVA with Fisher LSD *post-hoc* test for between group significance. Bars on figures indicate mean ± standard error of mean (SEM) for each analyses. Significant changes were shown for expression of SYK, LAMP-1, CD68, MME, and TGFB1.

Data for selected key genes of interest involved in CSF-1R signaling, inflammation, cell division and Aβ metabolism are also presented in graphical form in Figures 4, 5. The data for these figures are presented as corrected FPKM rather than relative expression levels to highlight the variability in expression between the different microglial isolates, and to demonstrate the expression levels of these different genes of interest. Due to the small numbers of cases, statistical significance was not reached for a number of the selected genes, but these genes are presented to reflect trends in changes in genes associated with activation phenotypes.

There was a trend for increased expression of CD14, the LPS receptor, a classical activation marker, but no significant change in MRC1 or CD163, considered alternative activation markers, or for CD40 an established proinflammatory activation marker. There were significant increases in TLR-2 and CCL2, activation-associated markers, but decreased expression of HLA-DR and TLR5 (data shown in Figure 4). Considering the genes associated with CSF-1R signaling (Figure 5), there were no changes in expression of CSF-1R, CSF-1, or the alternate IL-34 receptor PTPRZ1 with IL-34 or CSF-1 treatments. It was noticeable PTPRZ1 was expressed at very low levels in microglia compared to CSF-1R. This would suggest it has a minor role in IL-34 signaling in these types of cells. Expression of IL-34 mRNA was not detectable in any of the microglial samples. This is consistent with findings for rodent microglia (Mizuno et al., 2011). Both cytokines significantly downregulated expression of SYK, an essential signaling intermediate for many inflammatory pathways. The genes coding microglial proliferation markers PU.1 (SLI1) and Ki67 (MKI67) showed only small amounts of upregulation or downregulation. ICAM-1, an activation marker, showed a small degree of upregulation, while TREM-2 showed a degree of downregulation. This is consistent with recent data showing TREM-2 expression by human microglia being upregulated by alternative activation cytokines and downregulated by proinflammatory cytokines (Owens et al., 2017). One of the key issues for this study was how does CSF-1R activation affect genes associated with Aβ phagocytosis and degradation. The lysosomal-associated proteins TFEB, LAMP-1, and CD68 were all significantly downregulated by IL-34 and CSF-1 stimulation, as was expression of the Aβ degradative enzyme neprilysin (gene MME) (Figure 5). A slight increase in expression of IDE mRNA was detected. There was a significant increase in expression of the anti-inflammatory cytokine transforming growth factor (TGF) β1 with IL-34 and CSF-1 treatment (Figure 5).

**Figure 6:**
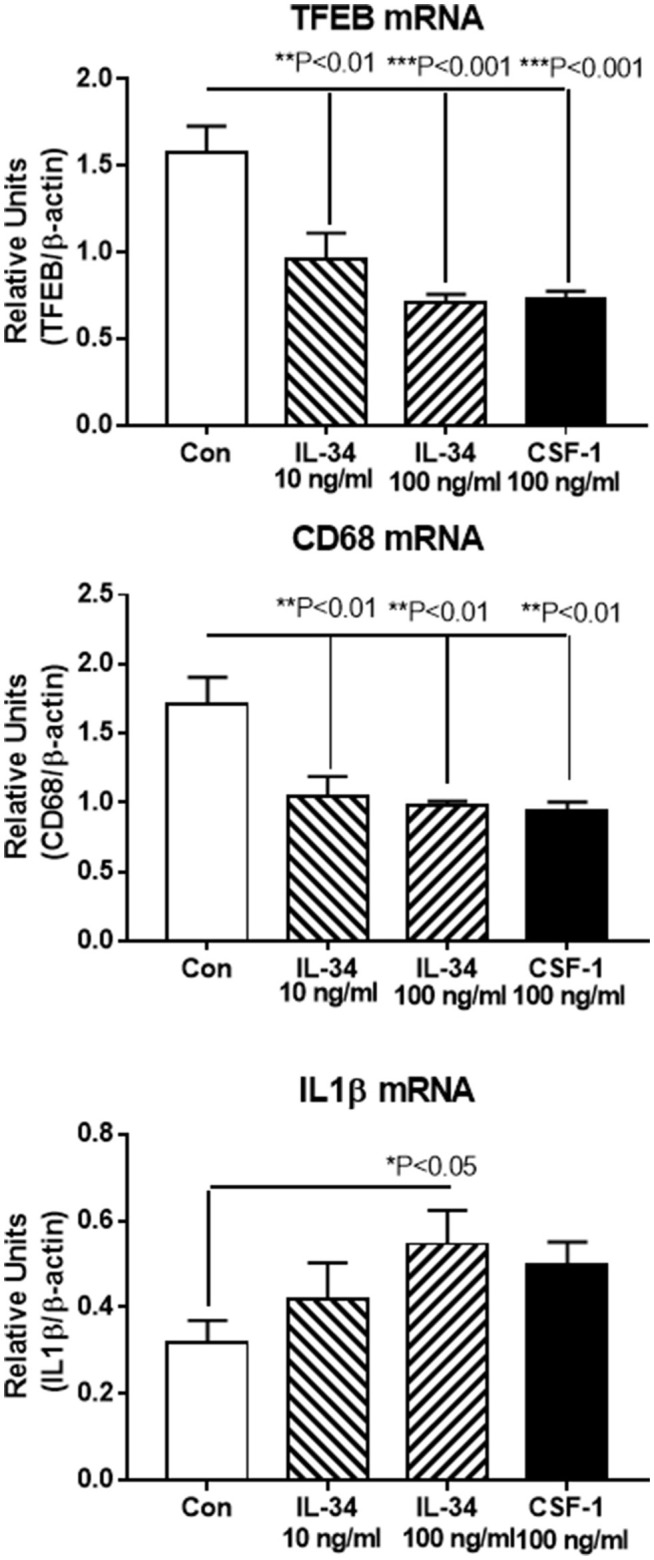
Real time PCR validation of key genes showing similar patterns of upregulation or downregulation as revealed by RNA sequencing data. Transcription factor EB (TFEB) and CD68 are both lysosomal-associated genes involved in Aβ phagocytosis and metabolism. IL-1β is a classical proinflammatory cytokine. Results represent mean ± SEM of triplicate determinations for each indicated treatment. Statistically significant differences with treatments were as indicated.

Real time PCR was used to validate expression of key genes in separate microglial samples. We focused on IL-1β, an established proinflammatory cytokine, and compared the results with TFEB and CD68, two lysosomal-associated proteins whose function are involved in Aβ phagocytosis and degradation. Our results show that the expression of these genes followed the patterns of upregulation and downregulation observed for RNA-seq (Figure 6).

### Effect of IL-34 on microglial activation, Aβ metabolism, and cell proliferation

**Figure 7:**
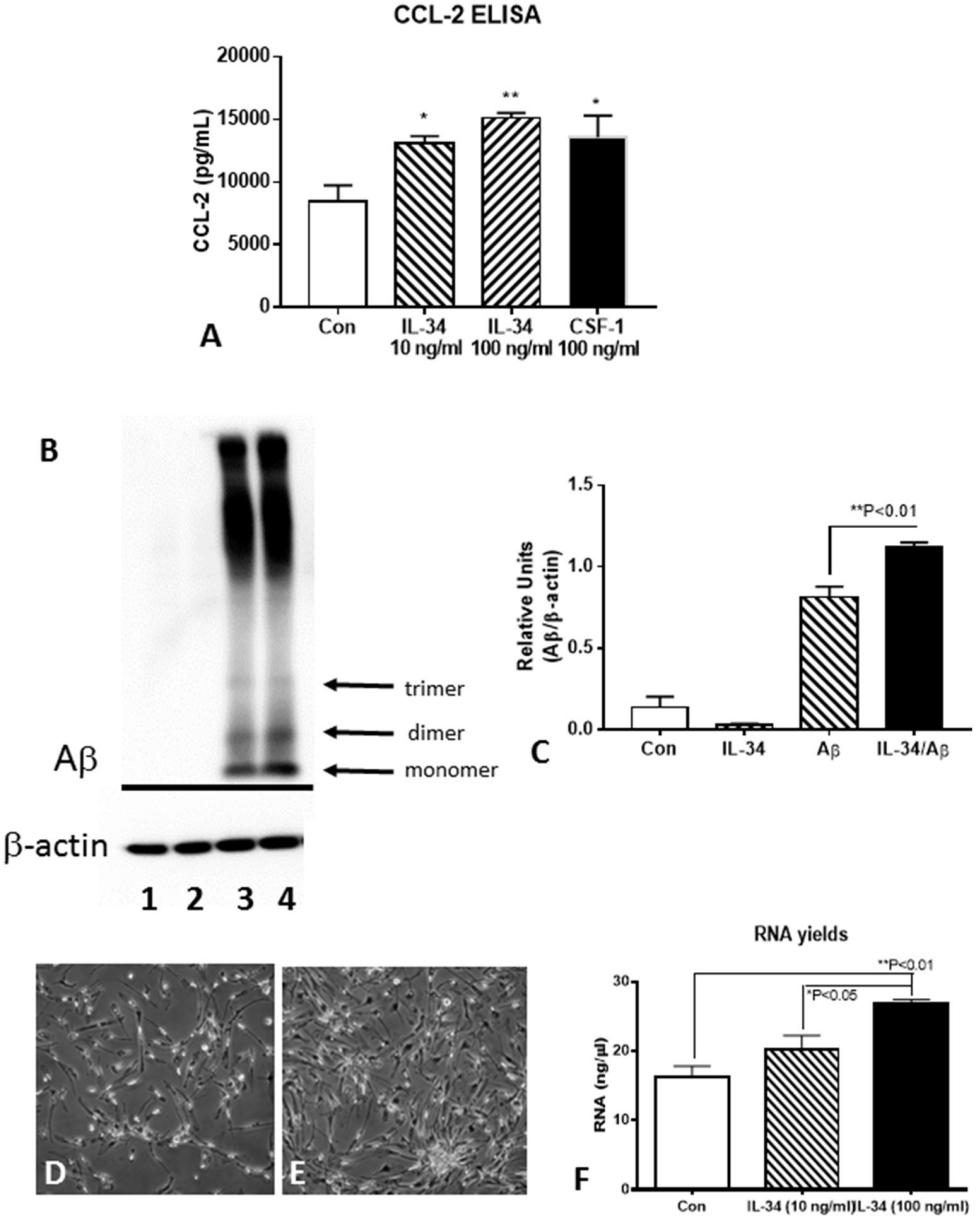
Effect of IL-34 on microglial functional properties. **(A)** ELISA measurements of CCL-2 from control, IL-34, and CSF-1 stimulated microglia. **(B,C)**. Effect of IL-34 on levels of Aβ (42) (1 μM) in treated microglia. **(B)** Representative western blot showing pattern of immunoreactivity for aggregated Aβ in treated microglia for 24 h. **(C)** Quantification of Aβ present in microglia after 24 h. Lane 1. Control untreated microglia: Lane 2. IL-34-treated microglia: Lane 3. Aβ (1 μM) treated microglia: lane 4 IL-34, and Aβ-treated microglia. **(D–F)** IL-34 effective at inducing significant cell division in human postmortem microglia. **(D)** Microglia treated without IL-34 for 4 days (representative field). **(E)** Microglia treated with IL-34 (100 ng/ml) showed significant increase in numbers. **(F)** Effect of IL-34 on total RNA yield of microglia treated for 4 days.

Functional assays were carried out using IL-34-stimulated microglia to determine the effects on activation, Aβ metabolism or cell proliferation (Figure 7). Using CCL-2 as a marker of inflammation, increased secretion of this chemokine was detected by ELISA with IL-34 treatments (Figure 7A). To determine whether IL-34 treatment affected Aβ uptake and degradation, a western blot method was used. Cells were pretreated with IL-34 for 2 h before addition of 1 μM of aggregated (fibril and oligomeric) Aβ42. This method would reflect steady-state intracellular levels of Aβ and values reflective of phagocytosis and degradation over the 24 h time period of analysis. The analysis showed that total levels of immunoreactive Aβ were actually increased in the IL-34 treated cultures (Figures 7B,C). This finding is consistent with our observations that expression for key lysosomal and Aβ enzymes are downregulated following IL-34 treatment.

To determine how effective IL-34 alone was at inducing cell division of human postmortem microglia, we treated cultures with IL34 at two doses (10 and 100 ng/ml) for 4 days. In preliminary experiments, we showed that this dose was effective at inducing cell division provided there was 0.5–1% fetal bovine serum (FBS) in the medium. In the absence of FBS, cell division was not apparent with IL-34 treatments. Significant cell division could be visible after 4 days of treatment (Figure 7D compared to Figure 7E). This was confirmed by measuring total yield of RNA from treated cultures at 4 days (Figure 7F). In our experiments, we had not observed significant effect of CSF-1 alone on microglial proliferation, but a mixture of CSF-1 and IL-34 had a synergistic effect resulting in significant cell division (unpublished data).

## Discussion

This study contained two separate components with the overall goal of assessing the expression and function of IL-34 in AD brains, and its effects on human aged brain-derived microglia. The studies using human brain samples reported in this paper showed a different pattern of expression of IL-34 compared to CSF-1 in AD. In one series of samples, there were decreased IL-34 mRNA expression and protein levels of IL-34 in AD brains, while there were increased expression of CSF-1 and CSF-1R mRNA in AD. In the second series of samples, there was not a significant change in IL-34 mRNA in the AD samples, while increased expression of CSF-1 and CSF-1R mRNA was confirmed. Using histological data that describes the severity of AD plaque and tangle pathology in each case, we showed significant correlations in expression of CSF-1 and CSF-1R with amounts of AD pathology, but no correlation between these measures and IL-34 mRNA expression. During the course of these studies, it was reported similarly that CSF-1R and CSF-1 mRNA levels were increased in AD temporal cortex, but no change in IL-34 mRNA levels (Olmos-Alonso et al., 2016). By contrast, they detected increased CSF-1, CSF-1R, and IL-34 mRNA expression in APP/PS1 plaque developing mice compared to wild type controls. Treatment of these APP/PS1 mice with the CSF-1R inhibitor GW2580 resulted in decreased expression of CSF-1 and CSF-1R mRNA but not IL-34. We showed no IL-34 mRNA expression in the RNA-seq dataset of microglial expressed genes, while CSF-1 and CSF-1R were expressed at high levels. The increased expression of CSF-1R, a microglial specific gene, and CSF-1 in conditions of inflammation in AD provide a mechanism for increased numbers of microglia to drive the inflammation (Akiyama et al., 1994). By contrast, the downregulation or no change of IL-34 expression indicates a potentially different function or mechanism of regulation. As IL-34 has only been localized to neurons in human brains, and not to glial cells, this might indicate a response to neurotoxicity or neuronal loss (Nandi et al., 2012; Wang et al., 2012). The samples used for this study were derived from cortical gray matter regions and the results indicate expression of both cytokines within this region.

Our findings have demonstrated the possibility of different types of responses by human elderly brain-derived microglia compared to other microglia types, particularly those derived from rodent. The gene expression studies by RNA-seq identified a stronger upregulation of proinflammatory genes than of anti-inflammatory genes, and also a significant downregulation of genes associated with Aβ phagocytosis and removal. We also attempted to define the phenotype of IL-34 and CSF-1 stimulated microglia by reference to the stimulation index of defined genes. Although there has been a lot of controversy about defining markers for proinflammatory or anti-inflammatory/alternative activation, especially for microglia, the panel of markers used showed a more proinflammatory activation rather than anti-inflammatory activation though the overlap between the groups is suggestive of a distinct phenotype between these groups (Murray et al., 2014). Of note was the strong induction of IL1B and related classical cytokines, though there was also increased expression of TGFB1, CCL4, CD209, and MRC1, considered alternative activation markers.

A central question also examined in this project was the proliferation of human postmortem brain microglia. These cells can be isolated from postmortem tissue samples, but the numbers obtainable are always limited. Attempts to obtained increased numbers of cells has proven difficult but is a desirable outcome as it permits more extensive experimentation. It was originally shown that CSF-1 produce some cell replication, but addition of granulocyte macrophage-CSF (GM-CSF) resulted in much greater proliferation (Lee et al., 1994). A recent paper showed significant induction of microglial proliferation with CSF-1 alone, but these studies employed microglial cultures with significant numbers of astrocytes, which could be producing additional growth factors such as GM-CSF (Smith et al., 2013). Our methods for isolating human microglia from postmortem brains can produce microglial cultures with purities of >99% (Walker et al., 2006); these cultures are generally not responsive to CSF-1 alone. Expression of the gene for GM-CSF (CSF-2) was present at very low or undetectable levels in the control and stimulated microglia cultures used in this study. A recent paper observed a high degree of proliferation of postmortem microglia treated with GM-CSF combined with a commercial preparation of microglial-media supplement, which likely included significant amounts of IL-34 (Guo et al., 2016). Our experiments focused on IL-34 as a human postmortem microglial growth factor and showed IL-34 alone induced significant amounts of cell division (example Figures 7D–F). High doses of IL-34 (100 ng/ml) were needed to obtain significant cell division. We found that IL-34 effects on microglia were not revealed in the absence of FBS in the culture media. We have confirmed that combining GM-CSF and IL-34 resulted in enhanced cell division of postmortem microglial cultures, though GM-CSF has a much stronger proinflammatory activation effect than CSF-1 and IL-34 (unpublished data).

The significance of altered CSF-1R signaling to microglial survival and activation has been highlighted by a series of studies involving administration of CSF-1R inhibitor agents to experimental rodents. Studies by one group used two related CSF-1R inhibitors, PLX3397, or PLX5622 administered in the animal feed, produced almost complete ablation of microglia from the animal brain at the highest dose (Elmore et al., 2014, 2015; Rice et al., 2015, 2017; Spangenberg et al., 2016). PLX5622 was used in subsequent experiments as it was more selective to CSF-1R tyrosine kinase inhibition. Ablation of microglia appeared to have beneficial consequences in AD rodent models. 5x FAD mice treated with PLX3397 prevented neuronal and dendritic spine loss even though there was no significant changes in the amounts of amyloid plaque pathology (Spangenberg et al., 2016). It was observed using 3xTgAD mice that treatments with CSF-1R inhibitor prevented accumulation of microglia around plaques (Dagher et al., 2015). Similar findings were reported by another group using the CSF-1R inhibitor GW2580 (Olmos-Alonso et al., 2016), where treated APP/PS1 animals showed significant improvement in the T-maze cognition test even though there were no significant differences in Aβ levels. The removal of microglia appeared to have beneficial effects on other neurodegenerative disease models, including stroke, cranial irradiation, toxin induced neurotoxicity, and amyotrophic lateral sclerosis (Rice et al., 2015, 2017; Acharya et al., 2016; Martinez-Muriana et al., 2016). Most studies using CSF-1R inhibitors have demonstrated therapeutic benefits of removal of microglia from rodent brains. This is surprising as other findings have shown that proinflammatory-activated microglia were needed for the efficient phagocytosis and removal of amyloid, which many believe is essential for effective AD treatment (Herber et al., 2007; Chakrabarty et al., 2010).

There are still many unanswered questions about the role of microglia in propagating AD pathology in human subjects. If removal of microglia from brain does not lead to enhanced pathology due to the increased accumulation of Aβ plaques, but does lead to reduced neurotoxicity, synaptic damage, and tau pathology due to decreased neuroinflammation, a reassessment of Aβ reduction as the primary therapeutic targets for AD might be needed. One unexplored area that we have tried to address and may be key to understanding the interrelationship of microglia to AD could be the effects of aging on microglial function. A recent paper showed that media from microglial cultures of young mice could supplement the phagocytosis ability for Aβ of microglia from old animals (Daria et al., 2017). Their finding suggest GM-CSF was the required agent to bring about this property. Our findings appear to differ from those using rodent microglia. Is this due to species difference or age difference? Further studies on the role of microglial age, particularly using human microglia from aged brains, in relation to their functional phenotypes could be informative for understanding of neuroinflammation in the aging brain.

## Author contributions

DW conceived study, developed experimental design, carried out data collection and analysis, prepared manuscript. TT carried out data collection and analysis. LL aided in experimental design, data collection, provided research materials, and aided in manuscript preparation.

### Conflict of interest statement

The authors declare that the research was conducted in the absence of any commercial or financial relationships that could be construed as a potential conflict of interest.
